# Natural-Product-Inspired Approaches for Cancer Diagnosis and Therapy

**DOI:** 10.3390/pharmaceutics14091884

**Published:** 2022-09-06

**Authors:** Eun Sook Lee, Jung Min Shin

**Affiliations:** 1 Safety Measurement Institute, Korea Research Institute of Standards and Science (KRISS), 267 Gajeong-ro, Yuseong-gu, Daejeon 34113, Korea; 2 Division of Biotechnology, Convergence Research Institute, DGIST, 333 Techno Jungang-Daero, Daegu 42988, Korea

In recent years, new methods of cancer diagnosis and therapy have emerged as promising options for fighting cancer. For example, biocompatible polymer-based materials, metal-phenolic hybrid networks, and inorganic nanoparticles are available for in vivo applications, such as tumor-specific diagnosis, targeted drug delivery, and immunotherapy. However, considerable limitations remain, such as off-target effects and the low therapeutic index, as well as low in vivo availability which restricts wider applications. For enhanced cancer theranostics, natural-product-inspired approaches might provide pointers for overcoming some of the obstacles in conventional techniques that deal with the high-resolution tumor-tissue-specific imaging, drug-delivery efficiency, and anticancer activity.

This Special Issue highlights recent advances in natural-product-inspired cancer diagnosis and therapy approaches. Nature-inspired materials, including potent compounds, extracts, and nano-formulated materials, were comprehensively considered in this Special Issue.

The publication by Lee et al. [1] revealed the role of hispolon on LPS-induced RAW264.7 cells; hispolon is a potent anti-inflammatory agent which can be isolated from mushrooms. The authors explore the immunomodulatory effect on RAW264.7 cells and lymphocyte proliferation using splenocytes isolated from mitogen-/alloantigen-treated mice. Hispolon showed an anti-inflammatory effect on LPS-induced RAW264.7 cells, with inhibition of reactive oxygen species (ROS) and nitrogen species (RNS). Additionally, it lessened the sulfhydryl content in both cell-free systems and RAW264.7 cells, implying its potential as an anti-inflammatory agent. The author also examined the cytokine level after hispolon treatment of LPS-induced RAW264.7 cells. Upon treatment with hispolon, the decreased levels of cytokines, including IL-6 and TNF-α, provided evidence for a significant anti-inflammatory effect. Furthermore, hispolon inhibited lymphocyte proliferation, suggesting its potential for mitogen-/alloantigen-treated splenocytes. Therefore, hispolon is a potential therapeutic agent for inflammatory immune diseases. These findings showing the immunomodulatory effect of hispolon on macrophages and lymphocytes provide evidence for the potential applications of hispolon.

The publication by Shipunova et al. [2] developed HER2-targeting silver nanoparticles (AgNPs) for the photothermal treatment of cancer. They produced 35 nm HER2-targeted silver nanoparticles by grind synthesis using *Lavandula angustifolia* Mill extract. The authors equipped the nanoparticle with the anti-HER2 affibody Z_HER2:342_. The HER2-targeted AgNPs showed a hypothermic effect in combination with light radiation treatment. The in vitro internalization of the HER2-targeted AgNP in HER2-overexpressing cells, as revealed by confocal microscopy and flow cytometry, signals the successful binding of the nanoparticle to HER2-overexpressing cells. Light eradiation successfully produced ROS with a cytotoxic effect of the HER2-targeted AgNPs. Using bioluminescent imaging, in vivo tumor growth inhibition upon treatment with HER2-targeted AgNPs was demonstrated to prevent metastatic spread after treatment. Therefore, the HER2-targeted AgNPs possess high potential as agents for photothermal therapeutic treatments of HER2-overexpressing cancers.

The publication by Hsu et al. [3] reported an antitumor effect of carotenoid extract and nanoemulsion from sweet potato peel against murine breast cancer cells in a tumor-bearing mice model. They fabricated a nanoemulsion with PEG using the sonication method. The nanoemulsion was 15.7 nm in diameter and had a −69.8 mV zeta potential. The fabricated nanoemulsion possessed high stability at 25 °C (90 days) and 100 °C (2 h). The anti-proliferation activity of the nanoemulsion was found to be superior to that of the carotenoid extract due to its structure. In the tumor-bearing mice model, the administration of the nanoemulsion reduced tumor volume by 36.1% compared with the control group. Upon nanoemulsion treatment, the concentration of VEGF in mouse serum decreased considerably, signaling its effect on tumor-formation-induced MCF-7 cancer cells. The authors suggest that the antitumor therapeutic efficacy of the nanoemulsion might be due to the synergistic effect of various carotenoid compositions in nanoemulsion compared with carotenoid extract.

In the publication by Nguyen et al. [4], the authors demonstrated that nootkatone suppresses the stemness of MCF-7/SC human breast cancer stem cells. Nootkatone, a natural compound produced in grapefruit, is widely used to stimulate energy metabolism. They first isolated MCF-7/SC cells from MCF-7 cells using CD44^+^ and CD24^−^ markers, which exerted higher tumorigenic ability than MCF-7 cells. Then, they demonstrated that MCF-7/SC cells utilized glycolysis for energy production. When the authors treated nootkatone with MCF-7/SC, it inhibited cell proliferation, impaired glucose metabolism, and reduced the stemness of MCF-7/SC. In summary, nootkatone has potential as an agent for treating breast cancer by modulating the stemness of breast cancer stem cells.

The publication by Duarte et al. [5] explored the uses of honeybee venom to treat cancer cells (HT-29 as the colon cancer model and MCF-7 as the breast cancer model) in combination with the central nervous system (CNS) drugs 5-fluorouracil (5-FU) and doxorubicin (DOX). The authors demonstrated that a sample of Portuguese honeybee venom showed a superior cytotoxic effect in MCF-7 cells compared with HT-29 cells. Additionally, a combinatorial treatment of honeybee venom and repurposed CNS drugs showed significant anticancer effects even in lower concentrations. This study explored the combinatorial potential of honeybee venom with CNS drugs for a synergistic cytotoxic effect in HT-29 cells and MCF-7 cells.

Tarar et al. [6] utilized sinigrin, which is abundant in cruciferous vegetables. The authors designed myrosinase-overexpressed adenocarcinoma cells that hydrolyzed the sinigrin and produced allyl isothiocyanate, which subsequently induced cell apoptosis. The myrosinase-tethered adenocarcinoma cells were prepared using a biotin-streptavidin system between biotinylated cells and myrosinase-core streptavidin fusion protein, which was obtained through bacterial expression. Synthesized fusion proteins were evaluated using SDS-PAGE and Western blot; in addition, the anticancer effect and apoptosis were analyzed in vitro. The overall experimental results suggest that sinigrin can be used as a promising prodrug, and that myrosinase sinigrin therapy has potential as a strategy for in situ cancer eradication.

The publication by Inbaraj et al. [7] demonstrated the preparation of nanocomposites composed of resveratrol in grape skin and showed their inhibition effects on pancreatic cancer cells. The authors note that the extraction of resveratrol in grape skin, discarded as a by-product of grape juice, has merit from the viewpoint of harnessing food waste in the food industry. To improve the biological activity of resveratrol, the authors synthesized two types of nanocomposites—nanoemulsions and gold-resveratrol nanoparticles—and compared the inhibition effects in vitro. Furthermore, the expression of proteins in relation to apoptosis was evaluated; finally, in vivo applications were proposed.

In the work of Platella and Ghirga et al. [8], five chemotypes which are capable of targeting the telomeric G-Quadruplex were selected from the highly diverse library of natural compounds. The authors implemented a docking-based virtual screening followed by G-Quadruplex in a controlled-pore glass assay. The experimental results from circular dichroism, fluorescence spectroscopy, and molecular dynamics simulations showed the affinity and selectivity of those compounds with G-Quadruplex models. In addition, biological studies, including viability assay, immunofluorescence and fluorescence in situ hybridization assay, clonogenic assay, and Western blot, were carried out to determine the anticancer activity on both cancerous and normal cells by stabilizing the telomeric G-Quadruplex. These selected compounds can be utilized for the novel design of anticancer drugs.

The development of anticancer drugs based on the sources derived from plants, marine organisms, and animals, especially reptiles, was reviewed by Prof. Jin Woong Chung and coworkers [9]. In particular, the bioactive components from the reptiles were classified into four categories, as follows: extracts, crude peptides, sera and bile, and venom. Furthermore, the therapeutic mechanisms were discussed. The authors highlighted the screening system for the identification of reptile-derived components in combination with various drug-delivery systems to improve the therapeutic effects.

With this Special Issue, we hope to highlight the promising potential of natural products from diverse natural-sources-inspired materials for various biomedical applications, such as cancer diagnosis and therapy.

## References

[1] Lee E.K., Koh E.M., Kim Y.N., Song J., Song C.H., Jung K.J. (2022). Immunomodulatory Effect of Hispolon on LPS-Induced RAW264.7 Cells and Mitogen/Alloantigen-Stimulated Spleen Lymphocytes of Mice. Pharmaceutics.

[2] Shipunova V.O., Belova M.M., Kotelnikova P.A., Shilova O.N., Mirkasymov A.B., Danilova N.V., Komedchikova E.N., Popovtzer R., Deyev S.M., Nikitin M.P. (2022). Photothermal Therapy with HER2-Targeted Silver Nanoparticles Leading to Cancer Remission. Pharmaceutics.

[3] Hsu H.-Y., Chen B.-H. (2022). A Comparative Study on Inhibition of Breast Cancer Cells and Tumors in Mice by Carotenoid Extract and Nanoemulsion Prepared from Sweet Potato (*Ipomoea batatas* L.) Peel. Pharmaceutics.

[4] Nguyen Y.T.-K., To N.B., Truong V.N.-P., Kim H.Y., Ediriweera M.K., Lim Y., Cho S.K. (2022). Impairment of Glucose Metabolism and Suppression of Stemness in MCF-7/SC Human Breast Cancer Stem Cells by Nootkatone. Pharmaceutics.

[5] Duarte D., Falcão S.I., El Mehdi I., Vilas-Boas M., Vale N. (2022). Honeybee Venom Synergistically Enhances the Cytotoxic Effect of CNS Drugs in HT-29 Colon and MCF-7 Breast Cancer Cell Lines. Pharmaceutics.

[6] Tarar A., Alyami E.M., Peng C.-A. (2022). Eradication of Myrosinase-Tethered Cancer Cells by Allyl Isothiocyanate Derived from Enzymatic Hydrolysis of Sinigrin. Pharmaceutics.

[7] Inbaraj B.S., Hua L.-H., Chen B.-H. (2021). Comparative Study on Inhibition of Pancreatic Cancer Cells by Resveratrol Gold Nanoparticles and a Resveratrol Nanoemulsion Prepared from Grape Skin. Pharmaceutics.

[8] Platella C., Ghirga F., Zizza P., Pompili L., Marzano S., Pagano B., Quaglio D., Vergine V., Cammarone S., Botta B. (2021). Identification of Effective Anticancer G-Quadruplex-Targeting Chemotypes through the Exploration of a High Diversity Library of Natural Compounds. Pharmaceutics.

[9] Park S.Y., Choi H., Chung J.W. (2022). Reptiles as Promising Sources of Medicinal Natural Products for Cancer Therapeutic Drugs. Pharmaceutics.

